# Oxidative balance score and its association with chronic inflammatory airway diseases and mortality: a population-based study

**DOI:** 10.3389/fnut.2025.1541559

**Published:** 2025-01-22

**Authors:** Zhuanbo Luo, Shiyu Chen, Peixu Chen, Feng Qiu, Weina Huang, Chao Cao

**Affiliations:** Department of Respiratory and Critical Care Medicine, Key Laboratory of Respiratory Disease of Ningbo, The First Affiliated Hospital of Ningbo University, Ningbo, Zhejiang, China

**Keywords:** oxidative balance score, chronic inflammatory airway diseases, mortality, population-based study, NHANES

## Abstract

**Objective:**

The aim of this research was to explore the possible connection between combined Oxidative Balance Score (OBS) and the prevalence of chronic inflammatory airway diseases (CIAD), including asthma, chronic obstructive pulmonary disease (COPD), and chronic bronchitis, along with the mortality rate among individuals with CIAD.

**Methods:**

Data were gathered from the National Health and Nutrition Examination Survey (NHANES) 2013–2018 cycles. The Oxidative Balance Score (OBS) was calculated using 16 different nutrients and 4 different lifestyles, which was then categorized into four groups. The CIAD included individuals with self-reported asthma, chronic bronchitis, or COPD. Mortality data up to December 31, 2019, was obtained from the National Death Index. In cross-sectional studies, the association between OBS and the prevalence of total and specific CIAD was examined using multiple logistic regressions. Dose–response relationships were analyzed through restricted cubic spline regression (RCS). In prospective cohort studies, cumulative survival rates were determined using the Kaplan–Meier method and compared with log-rank tests. Multiple COX regressions were conducted to evaluate the relationship between OBS and all-cause as well as respiratory diseases mortality among participants with CIAD.

**Results:**

A total of 12,458 adults were enrolled in this study. The demographic characteristics of the study population revealed a mean age of 52.25 ± 15.8 years, 47.73% being male, and the majority identified as Non-Hispanic White (66.87%). We found that 20.26% of the participants were suffered from CIAD, followed by asthma (15.41%), chronic bronchitis (6.10%) and COPD (3.80%), respectively. The median OBS levels were 20.98 with a standard deviation of 0.17. After adjusting for all confounders, we found that the highest quartile of OBS was significantly associated with lower prevalence of total CIAD (OR = 0.71, 95% CI 0.64–0.81), asthma (OR = 0.62, 95% CI 0.52–0.73), chronic bronchitis (OR = 0.64, 95% CI 0.44–0.92), and COPD (OR = 0.48, 95% CI 0.31–0.77) compared to the lowest quartile. Additionally, a linear and inverse relationship was found between OBS and the incidence of various respiratory disorders. Kaplan–Meier survival analysis showed that individuals in the highest quartile of OBS had the lowest risk of both all-cause mortality (log-rank test *p* = 0.017) and respiratory diseases mortality (log-rank test *p* < 0.001). Furthermore, after adjusting for multiple factors, individuals in the fourth quartile continued to show a significantly reduced risk of all-cause mortality (HR = 0.71, 95% CI 0.55–0.93) and respiratory diseases mortality (HR = 0.53, 95% CI 0.43–0.74) in comparison to those in the lowest quartile of OBS levels.

**Conclusion:**

The findings revealed that a higher OBS was significantly linked to a decreased prevalence of total and specific CIAD, including asthma, chronic bronchitis, and COPD. Higher OBS levels were also associated with reduced mortality from both all causes and respiratory diseases among CIAD patients. These findings offer valuable information on the role of diet and lifestyle in preventing CIAD.

## Introduction

Chronic inflammatory airway diseases (CIAD), including asthma, chronic bronchitis, and chronic obstructive pulmonary disease (COPD), have posed a significant threat to public health for a long time (1). The effects of air pollution, tobacco exposure, and unhealthy habits on respiratory diseases have evident due to the rise of industrialization, urbanization, and an aging population, leading to a yearly rise in both the occurrence and death rate of many chronic respiratory illnesses. In 2017, chronic respiratory diseases ranked as the third highest cause of death following cardiovascular diseases and cancers, making up 7.0% of all deaths, with asthma and COPD being the most widespread chronic respiratory conditions (1). The 2019 global burden of disease data revealed more than 260 million asthma cases with inadequate control, leading to 455,000 deaths (2). Furthermore, there were 3.3 million deaths from COPD epidemic cases among a total of 212.3 million cases (3). Research has increasingly indicated that oxidative stress, triggered by lifestyle and dietary choices, may play a crucial role in the development and mortality of CIAD (4). However, the exact relationship between these factors remains to be fully understood.

Oxidative stress occurs when there is an unequal level of pro-oxidant and antioxidant activities in the body. This imbalance can result in oxidative harm, which forms the foundation for a range of conditions, including inflammatory diseases, aging, metabolic disorders, cancer, and more (5–7). In asthma, this imbalance between pro-oxidants and antioxidants is a contributing factor, with individuals who develop the condition often exhibiting increased oxidant production and decreased antioxidant defenses (8). The progression of COPD could also be facilitated by oxidative stress, which harms the DNA of cells, reduces anti-protease functions, and speeds up the fibrosis of small airways (9). Meanwhile, oxidative stress was found to induce immune cells to release large amounts of inflammatory mediators such as interleukin-6 and tumor necrosis factor-*α*, which may even further lead to the production of inflammatory storms and result in lung tissue damage and emphysema (10). In addition, oxidative stress may exert adverse effects such as cortisol hormone resistance and the development of airway hyperresponsiveness, involved in the mechanism of CIAD (11). In conclusion, a large number of studies have shown that a variety of oxidative stress factors, including but not limited to reactive oxygen species, nitrogen oxides and the imbalance of antioxidant activity, all play a key role in the pathogenesis of CIAD (12). Furthermore, studies have shown that oxidative stress is linked to various lifestyle and dietary factors, which can be assessed using the Oxidative Balance Score (OBS) (13). Certain lifestyle habits, such as smoking and excessive drinking, as well as being overweight and consuming inadequate doses of vitamins A, C, and E, are pro-oxidant factors. In contrast, a balanced diet rich in antioxidants, maintaining a healthy weight, abstaining from smoking and alcohol, and engaging in regular physical activity are antioxidant factors (13, 14). Nowadays many studies have demonstrated a potential link between OBS and a variety of diseases, such as osteoarthritis, kidney disease, cardiovascular disease, depression, diabetes, and certain cancers (15–19). However, we did not find any reports of an association between CIAD and OBS, and the relationship between the two is currently unclear.

Therefore, to address this gap, our study utilized data from 12,458 participants aged 20 and older from the national health and nutrition examination survey (NHANES) 2013–2018 database to investigate the potential correlation between OBS and CIAD prevalence including asthma, chronic bronchitis, and COPD. Furthermore, we also conducted an investigation into the relationship between OBS and all-cause and respiratory diseases mortality among individuals with CIAD in order to offer scientific evidence and recommendations regarding the management and prevention of respiratory diseases by changing diet and lifestyle.

## Methods

### Study design and population

The National Health and Nutrition Examination Survey (NHANES), carried out by the Centers for Disease Control and Prevention (CDC), aims to evaluate the health status and nutritional well-being of the US population through a combination of interviews, physical examinations, and laboratory tests. The survey collects comprehensive data on population characteristics, eating habits, medical history, and biological markers. This valuable dataset serves as a critical resource for tracking health trends, pinpointing risk factors for disease, and shaping public health initiatives. The entire NHANES dataset is publicly available and can be freely downloaded from the CDC website^1^. The study was reviewed and approved by the National Center for Health Statistics Research Ethics Review Board, and all participants provided informed consent in writing.

**Figure 1:**
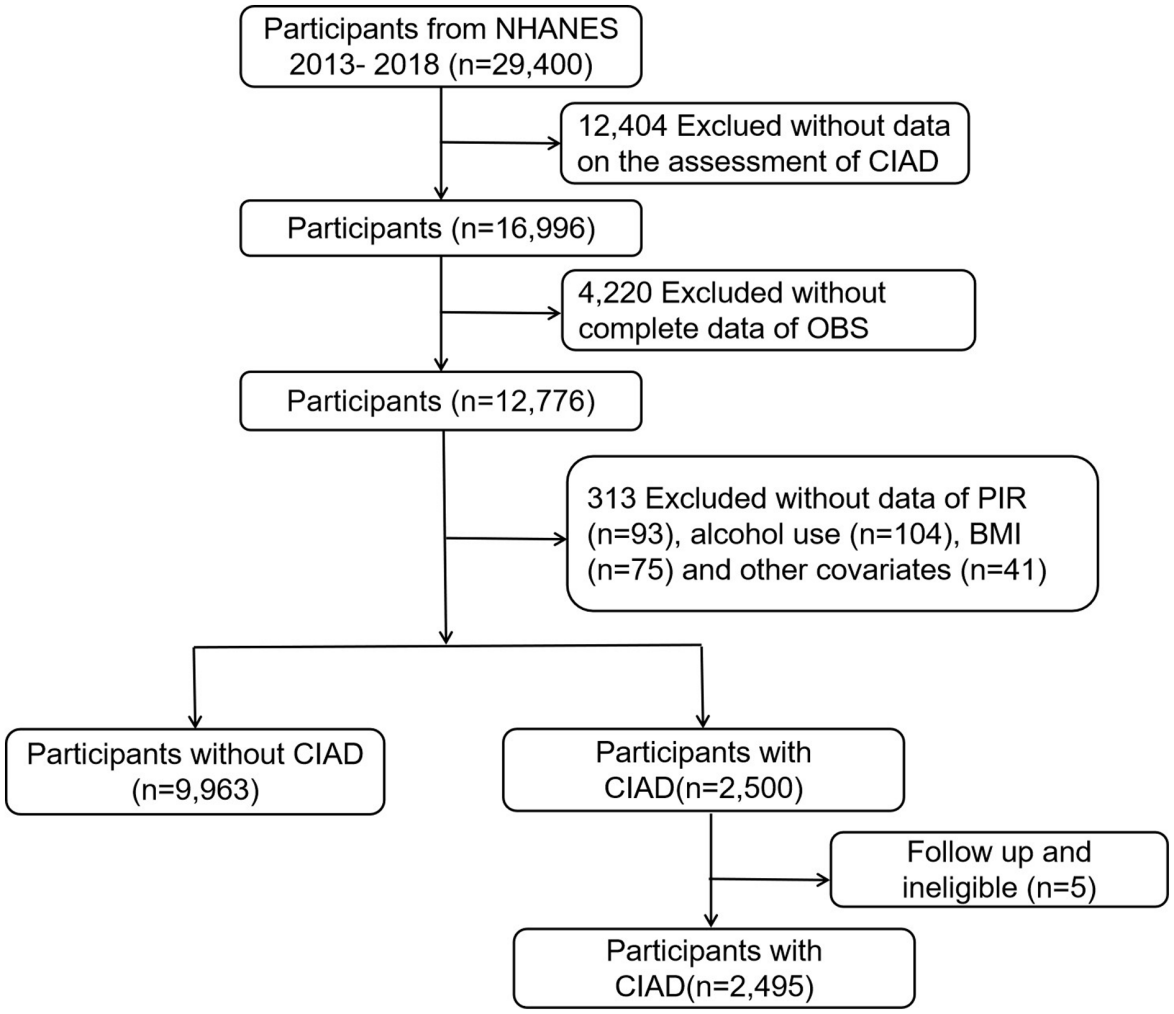
Flowchart of the study participants.

Using data from the NHANES 2013–2018 cycles, this study included participants aged 20 and above with complete information on diet and lifestyle for calculating OBS and CIAD assessment. Criteria for exclusion comprised those with missing CIAD assessment data, and those with missing OBS component data, and those with missing mortality data or other covariates. This process yielded a study group of 12,458 participants, consisting of 9,963 without CIAD and 2,495 participants with CIAD. Refer to Figure 1 for a detailed visual representation of the selection process.

### Definition of OBS

The Oxidative Balance Score (OBS) is determined by analyzing 16 different nutrients and 4 distinct lifestyles as outlined in 10 (13). These nutrients encompass fiber, carotene-retinol equivalent (RE), riboflavin, niacin, total folate, vitamin B6, vitamin B12, vitamin C, vitamin E, calcium, magnesium, zinc, copper, selenium, iron, and total fat. During the initial 2 days of interviews, these nutrients were evaluated without any additional dietary supplements. Among the dietary components contributing to OBS, iron and total fat were classified as pro-oxidants, while the remaining 14 nutrients were deemed antioxidants. In terms of lifestyle factors, physical activity was identified as having an antioxidant effect, whereas factors such as alcohol consumption, body mass index (BMI), and serum cotinine were considered pro-oxidants. Thus, the OBS comprised 15 antioxidant elements and 5 pro-oxidant elements (Table 1). BMI was calculated by dividing weight by height squared (kg/m^2^), and weekly metabolic equivalent (MET) values were derived from household interviews regarding leisure-time activities over the past month.

**Table 1:** Oxidative balance score assignment scheme.

OBS components	Property	Male			Female		
		0	1	2	0	1	2
Dietary components							
Dietary fiber (g/d)	Antioxidant	<13.6	13.6–21.25	≥21.25	<11.75	11.75–17.7	≥17.7
Carotene (RE/d)	Antioxidant	<106.96	106.96–382.79	≥382.79	<121.96	121.96–439.79	≥439.79
Riboflavin (mg/d)	Antioxidant	<1.77	1.77–2.54	≥2.54	<1.41	1.41–2	≥2
Niacin (mg/d)	Antioxidant	<22.83	22.83–32.13	≥32.13	<16.49	16.49–23.32	≥23.32
Total folate (mcg/d)	Antioxidant	<335.5	335.5–499	≥499	<264	264–393	≥393
Vitamin B6 (mg/d)	Antioxidant	<1.75	1.75–2.55	≥2.55	<1.32	1.32–1.92	≥1.92
Vitamin B12 (mcg/d)	Antioxidant	<3.6	3.6–6.13	≥6.13	<2.63	2.63–4.57	≥4.57
Vitamin C (mg/d)	Antioxidant	<44.2	44.2–103.9	44.2–103.9	<43.5	43.5–93.25	43.5–93.25
Vitamin E (ATE) (mg/d)	Antioxidant	<6.04	6.04–9.39	≥9.39	<5.2	5.2–8.11	≥8.11
Calcium (mg/d)	Antioxidant	<756	756–1,140	≥1,140	<629.5	629.5–931.5	≥931.5
Magnesium (mg/d)	Antioxidant	<260.5	260.5–365	≥365	<217.5	217.5–296	≥296
Zinc (mg/d)	Antioxidant	<9.77	9.77–14	≥14	<7.38	7.38–10.32	≥10.32
Copper (mg/d)	Antioxidant	<1.06	1.06–1.51	≥1.51	<0.89	0.89–1.25	≥1.25
Selenium (mcg/d)	Antioxidant	<102.75	102.75–142.6	≥142.6	<74.7	74.7–105.3	≥105.3
Iron (mg/d)	Prooxidant	≥18.23	12.72–18.23	<12.72	≥14.25	9.88–14.25	<9.88
Total fat (g/d)	Prooxidant	<9.88	67.74–98.25	<67.74	≥75.04	51.71–75.04	<51.71
Lifestyle components							
Physical activity (MET-minute/week)	Antioxidant	<720	720–1988	≥1988	<540	540–1,440	≥1,440
BMI (kg/m^2^)	Prooxidant	≥29.52	25.3–29.52	<25.3	≥30.52	24.7–30.52	<24.7
Cotinine (ng/mL)	Prooxidant	≥0.5	0.02–0.5	<0.02	≥0.07	0.01–0.07	<0.01
Alcohol (g/d)	Prooxidant	≥30	0–30	<0	≥15	0–15	<0

OBS, oxidative balance score; RE, retinol equivalent; ATE, alpha-tocopherol equivalent; MET, metabolic equivalent; BMI, body mass index.

The points assigned to OBS components included a range of scores from 0 to 2 allocated to antioxidants and pro-oxidants, segmented into tertiles from the first to the third. In the context of alcohol consumption, non-drinkers, moderate drinkers (up to 15 g/day for women and 30 g/day for men), and excessive drinkers (over 15 g/day for women and 30 g/day for men) were assigned scores of 2, 1, and 0, respectively. Similarly, other components were categorized into three tiers, with scores ranging from 0 to 2, adjusted for age-specific differences, akin to the classification based on alcohol consumption. Finally, the OBS is calculated by summing the points of each component, with higher scores indicating stronger antioxidant properties (13).

### Definition of CIAD

In this research, CIAD was defined as a self-reported asthma, COPD, and chronic bronchitis. Participants were inquired about previous diagnoses of asthma, COPD, or chronic bronchitis made by a healthcare provider. Those who confirmed having any of these diseases were classified as individuals with the respective condition. A positive test result for asthma, COPD, or chronic bronchitis indicated a positive diagnosis for CIAD. CIAD was identified as having at least one self-reported case of these respiratory conditions.

### Assessment of mortality

We carried out a prospective cohort analysis to evaluate how OBS affects mortality in individuals with CIAD. The study focused on both all-cause and respiratory disease mortality, including deaths from any reasons and respiratory illnesses, such as chronic lower respiratory diseases, influenza, and pneumonia. To gather mortality data, the study linked NHANES data with the National Death Index using a probabilistic matching method that relied on personal details such as name, date of birth, social security number, and gender. The follow-up duration was calculated from the NHANES interview date until the date of death or December 31, 2019, whichever came earlier.

### Covariates

To minimize potential biases, we included a range of covariates in our analysis. We gathered baseline data on participants through questionnaires and laboratory tests, covering factors such as age (<40, 40–60, or >60 years old), sex (male or female), educational level (more than high school, completed high school, or less than high school), racial and ethnic background (Mexican American, Non-Hispanic White, Non-Hispanic Black, other races), body mass index (BMI) (<25.0, 25.0–29.9, or >30.0 kg/m^2^) and energy intake (continuous). Furthermore, we also assessed socioeconomic status using the poverty-income ratio (PIR; the ratio of family’s income divided by poverty threshold that corresponds to the family size as defined by the US Department of Health and Human Services) and categorized it into three levels: <1.3, 1.3–3.5, and >3.5 (20). Marital statuses were grouped into married/living with partner, widowed/divorced, or never married. Self-reported smoking habits included never smoker (smoked fewer than 100 cigarettes in their lifetime), former smoker (smoked over 100 cigarettes but no longer smoke at all), and current smoker (had smoked over 100 cigarettes in life and currently smoke) (21). Drinking status was categorized as never drinker (had less than 12 drinks in their lifetime), former drinker (had at least 12 drinks in 1 year but did not drink in the last year, or had not drank in the last year but had at least 12 drinks in their lifetime), current heavier drinker [consuming three or more drinks per day for females, four or more drinks per day for males, or engaging in binge drinking (four or more drinks on same occasion for females, five or more drinks on same occasion for males) on 5 or more days per month], or current mild/moderate drinker (consuming two or fewer drinks per day for females, three or fewer drinks per day for males, or engaging in binge drinking on two or fewer days per month) (22, 23).

Medical status variables taken into account included hypertension, hyperlipidemia, cardiovascular disease (CVD) and diabetes mellitus (DM). Hypertension was defined as systolic blood pressure of 130 mmHg or higher, diastolic blood pressure of 80 mmHg or higher, or the use of medication to control blood pressure (24). Diabetes mellitus (DM) was diagnosed as a condition identified by a doctor or other medical provider, or glycated hemoglobin level of over 6.5%, or random blood glucose level of 11.1 mmol/L or higher, or two-hour oral glucose tolerance test (OGTT) blood glucose level of 11.1 mmol/L or higher, or the use of diabetes medication or insulin (24). Hyperlipidemia was defined as triglycerides of 200 mg/dL or higher, or total cholesterol of 200 mg/dL or higher, or LDL cholesterol of 130 mg/dL or higher, or low HDL cholesterol levels (less than 40 mg/dL for men or less than 50 mg/dL for women), or the use of medications to lower lipid levels (24). Individuals who reported being diagnosed by a doctor with conditions such as coronary heart disease, heart attack, congestive failure, angina or stroke were categorized as having cardiovascular disease (24).

### Statistical analysis

All statistical analyses adhered to the guidelines set forth by the NHANES for data analysis and reporting and took into consideration the stratified survey design factors. We categorized participants into four groups based on their OBS levels, ranging from the lowest level in Quartile 1 (Q1) to the highest level in Quartile 4 (Q4). Normally distributed continuous variables were presented as means and standard error (SE), while non-normally distributed continuous variables are shown as medians and interquartile ranges. Categorical variables were displayed as numbers (percentages). To identify significant differences between groups, the chi-square test and one-way ANOVA were utilized for categorical and continuous variables, respectively. Additionally, we used a multiple logistic regression model to determine the adjusted odds ratios (ORs) and 95% confidence intervals (CIs) for the relationship between OBS quartiles and the prevalence of total and specific CIAD. Three distinct models were built: Crude Model without adjustments; Model 1 was adjusted for gender, age, and ethnicity; and Model 2 further incorporated education level, family income-to-poverty ratio, smoking and drinking status, marital status, BMI, energy intake, and medical histories of hypertension, hyperlipidemia, cardiovascular disease and diabetes on the basis of Model 1 to provide a more comprehensive analysis. The association between OBS levels and inflammatory markers in adults was determined using multiple linear regression after adjusting for multiple factors. We calculated *β* values and corresponding 95% CIs.

To further investigate the dose–response curves of the relationship between OBS levels and the prevalence of total and specific CIAD, we utilized restricted cubic spline regression analysis with specific knots set at the 10th, 50th, and 90th percentiles of OBS levels. We also performed stratified analyses in various subgroups. In prospective cohort studies, the Kaplan–Meier method was used to estimate cumulative mortality rates from all causes and respiratory diseases, and log-rank tests were employed to compare survival rates among four groups of participants, categorized by quartiles of OBS levels. Moreover, multiple COX regressions were applied to estimate adjusted hazard ratios (HRs) and 95% CIs in relation to all-cause and respiratory disease mortality of participants with CIAD. All statistical analyses were performed using the R Project for Statistical Computing (version 4.3.3), with statistical significance defined as a two-sided *p*-value of less than 0.05.

## Results

### Baseline characteristics

The research analyzed data chosen from three consecutive two-year cycles of NHANES (2013–2018), with their baseline characteristics categorized by OBS quartiles summarized in Table 2.

**Table 2:** Weighted characteristics of the study population according to OBS quartiles.

Variable	Total (*N* = 12,458)	OBS				*P*-value
		Q1(*N* = 3,586)	Q2(*N* = 2,681)	Q3(*N* = 3,397)	Q4(*N* = 2,794)	
		[3,15]	[15,20]	[20,26]	[26,37]	
Age, %						<0.001
<40	3,870(33.37)	984(32.30)	839(33.37)	1,107(33.78)	940(33.93)	
40–60	4,507(39.12)	1,233(37.50)	935(37.97)	1,268(39.57)	1,071(41.15)	
>60	4,081(27.51)	1,369(30.20)	907(28.66)	1,022(26.65)	783(24.92)	
Sex, %						0.01
Female	6,501(52.27)	1787(49.98)	1,374(51.99)	1824(53.88)	1,516(52.88)	
Male	5,957(47.73)	1799(50.02)	1,307(48.01)	1,573(46.12)	1,278(47.12)	
Family PIR, %						<0.001
<1.3	2,620(20.44)	997(27.83)	586(21.86)	574(16.93)	463(16.58)	
1.3–3.5	5,396(44.05)	1,262(35.23)	1,068(39.84)	1,674(49.31)	1,392(49.81)	
>3.5	4,442(35.52)	1,327(36.94)	1,027(38.30)	1,149(33.75)	939(33.62)	
Body mass index, %						<0.001
<25.0 kg/m^2^	3,338(27.32)	700(19.31)	641(23.48)	962(27.94)	1,035(37.59)	
25.0–29.9 kg/m^2^	3,979(31.65)	1,114(30.08)	851(32.15)	1,105(31.23)	909(33.21)	
>30.0 kg/m^2^	5,141(41.03)	1772(50.61)	1,189(44.37)	1,330(40.82)	850(29.20)	
Race/ethnicity, %						<0.001
Mexican American	1743(8.03)	394(6.69)	385(7.94)	505(8.54)	459(8.81)	
Non-Hispanic Black	2,734(11.00)	1,146(17.96)	589(11.31)	618(8.96)	381(6.31)	
Non-Hispanic White	4,897(66.87)	1,310(61.90)	1,077(67.87)	1,371(67.64)	1,139(69.98)	
Other races	3,084(14.10)	736(13.45)	630(12.87)	903(14.86)	815(14.91)	
Marital status, %						<0.001
Married/living with partner	7,487(64.36)	1942(57.96)	1,595(63.83)	2,123(66.47)	1827(68.60)	
Never married	2,236(17.37)	690(19.58)	461(16.26)	587(16.40)	498(17.25)	
Widowed/divorced	2,735(18.27)	954(22.46)	625(19.91)	687(17.12)	469(14.15)	
Education level, %						<0.001
Completed high school	2,865(23.26)	1,003(30.28)	663(26.64)	719(21.44)	480(15.69)	
Less than high school	2,358(11.67)	897(17.59)	528(12.30)	546(9.51)	387(7.87)	
More than high school	7,235(65.08)	1,686(52.13)	1,490(61.07)	2,132(69.05)	1927(76.44)	
Smoking status, %						<0.001
Former	3,025(25.69)	875(24.88)	645(24.90)	841(27.23)	664(25.39)	
Never	7,122(56.98)	1729(47.50)	1,511(54.59)	2027(58.47)	1855(66.45)	
Now	2,311(17.33)	982(27.63)	525(20.51)	529(14.30)	275(8.16)	
Drinking status, %						<0.001
Current heavier drinker	2,660(21.19)	855(23.85)	576(21.47)	628(18.48)	601(21.51)	
Current light/moderate drinker	7,238(58.55)	1873(52.24)	1,560(58.22)	2,129(62.68)	1,676(59.85)	
Former	1,240(9.83)	416(11.62)	258(9.63)	300(8.85)	266(9.52)	
Never	1,320(10.43)	442(12.29)	287(10.68)	340(9.99)	251(9.12)	
Energy intake, kcal/day	2178.95 (1788.6, 2511.3)	1320.59 (1095.4, 1783.6)	1832.54 (1535.4, 2241.6)	2221.05 (1853.7, 2634.9)	2807.08 (2454.3, 3149.5)	<0.001
CVD						<0.001
No	11,062(91.06)	3,007(86.70)	2,370(91.21)	3,088(92.10)	2,597(94.00)	
Yes	1,396(8.94)	579(13.30)	311(8.79)	309(7.90)	197(6.00)	
DM						<0.001
No	9,976(84.67)	2,675(80.31)	2,104(82.54)	2,780(85.38)	2,417(89.88)	
Yes	2,482(15.33)	911(19.69)	577(17.46)	617(14.62)	377(10.12)	
Hypertension						<0.001
No	6,852(59.93)	1,694(53.43)	1,451(57.84)	1955(61.09)	1752(66.64)	
Yes	5,606(40.07)	1892(46.57)	1,230(42.16)	1,442(38.91)	1,042(33.36)	
Hyperlipidemia						0.07
No	4,135(33.46)	1,099(30.66)	891(33.25)	1,151(33.88)	994(35.58)	
Yes	8,323(66.54)	2,487(69.34)	1790(66.75)	2,246(66.12)	1800(64.42)	
COPD, %						<0.001
No	11,946(96.20)	3,347(93.38)	2,565(96.06)	3,291(96.79)	2,743(98.36)	
Yes	512(3.80)	239(6.62)	116(3.94)	106(3.21)	51(1.64)	
Asthma, %						0.03
No	10,559(84.59)	2,939(82.89)	2,281(84.56)	2,931(86.13)	2,408(87.50)	
Yes	1899(15.41)	647(17.11)	400(15.44)	466(13.87)	386(12.50)	
Chronic bronchitis, %						<0.001
No	11,684(93.90)	3,269(91.28)	2,517(93.53)	3,211(94.29)	2,687(96.28)	
Yes	774(6.10)	317(8.72)	164(6.47)	186(5.71)	107(3.72)	
CIAD, %						<0.001
No	9,963(79.74)	2,704(75.69)	2,152(79.26)	2,792(81.53)	2,315(82.03)	
Yes	2,495(20.26)	882(24.31)	529(20.74)	605(18.47)	479(17.97)	

OBS, Oxidative Balance Score; PIR, poverty income ratio; CIAD, chronic inflammatory airway disease; COPD, chronic obstructive pulmonary disease; CVD, cardiovascular disease; DM, diabetes mellitus.

The demographic characteristics of the study population revealed a mean age of 52.25 ± 15.8 years, 47.73% being male, and the majority identified as Non-Hispanic White (66.87%). The study found that 20.26% of the participants suffered from CIAD, followed by asthma (15.41%), chronic bronchitis (6.10%) and COPD (3.80%), respectively. The median OBS levels were 20.98 with a standard deviation of 0.17. In comparison to the individuals in the first quartile (Q1) of OBS, the participants in the fourth quartile (Q4) were more likely to be young to middle-aged females, to be Non-Hispanic White, to have higher levels of education and middle income level, to have lower BMI, to be married or living with partner, to have current light-to-moderate alcohol consumption, to be non-smokers and have a higher energy intake. Furthermore, the participants of Q4 had a lower prevalence of cardiovascular disease, diabetes and hypertension, as well as chronic inflammatory airway diseases compared to the participants of Q1.

### Prevalence of CIAD

**Figure 2:**
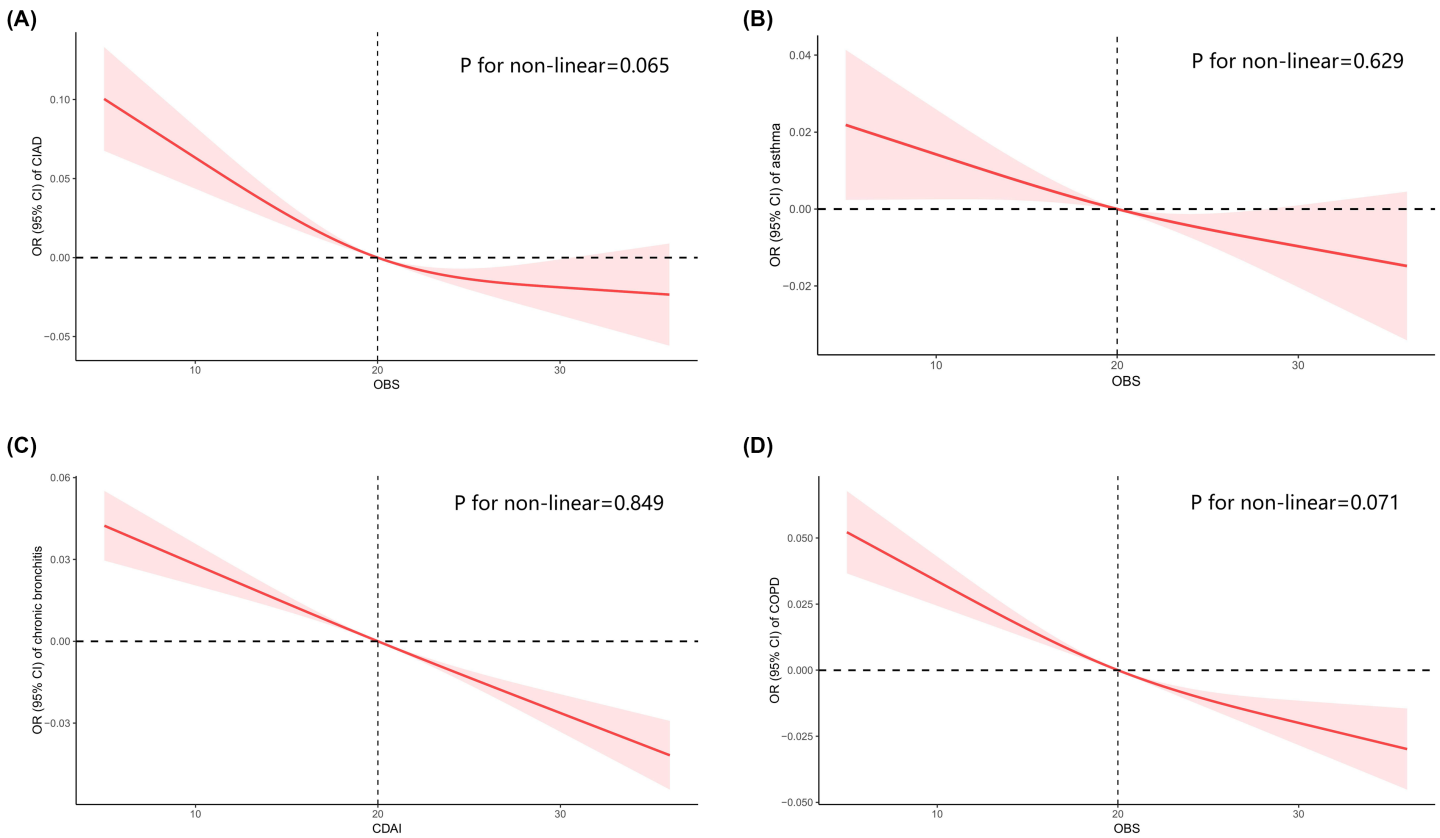
Restricted cubic spline analyses the association of Oxidative Balance Score (OBS) levels and the prevalence of total and specific chronic inflammatory airway diseases (CIAD) **(A)**, including asthma **(B)**, chronic bronchitis **(C)**, and COPD **(D)**. Adjusted for gender (female or male), age (<40, 40–60 or >60) and race (Mexican American, Non-Hispanic Black, Non-Hispanic White or other races); education level (completed high school, less than high school or more than high school), family income-to-poverty ratio (<1.3, 1.3–3.5 or >3.5), smoking status (never smoker, former smoker, or current smoker), drinking status (current heavier drinker, current light/moderate drinker, former drinker or never drinker), marital status (married/living with partner, never married or widowed/divorced), BMI (<25.0, 25.0–29.9 or >30.0 kg/m^2^), energy intake levels, histories of hypertension (yes or no), hyperlipidemia (yes or no), cardiovascular disease (yes or no) and diabetes (yes or no).

In this study, OBS was divided into quartiles, with the lowest quartile as the reference group, to investigate its relationship with the prevalence of total and specific CIAD (Table 3). The crude model indicated a negative association between OBS quartiles and the prevalence of both total and specific CIAD. This relationship remained significant even after adjusting for demographics such as age, gender, and race. Specifically, the third and fourth quartiles of OBS were linked to a reduced prevalence of total CIAD, asthma, chronic bronchitis, and COPD compared to the reference quartile in Model 1. After adjusting for all confounders, we found that the highest quartile of OBS was significantly associated with lower prevalence of total CIAD (OR = 0.71, 95% CI 0.64–0.81), asthma (OR = 0.62, 95% CI 0.52–0.73), chronic bronchitis (OR = 0.64, 95% CI 0.44–0.92), and COPD (OR = 0.48, 95% CI 0.31–0.77) compared to the lowest quartile in Model 2. Trend tests across all models underscored this relationship (all *p* for trend < 0.05), indicating a significantly reduced likelihood of both total and specific CIAD in participants with elevated OBS levels. Additionally, the linear and inverse relationship were found between OBS levels and the incidence of various respiratory disorders, including CIAD (*P* for non-linear = 0.065, Figure 2A), asthma (*P* for non-linear = 0.629, Figure 2B), chronic bronchitis (*P* for non-linear = 0.849, Figure 2C) and COPD (*P* for non-linear = 0.071, Figure 2D).

**Table 3:** Odds ratios (ORs) (95% CIs) of the prevalence of chronic inflammatory airway diseases (CIAD) according to quartiles of OBS among adults in NHANES 2013–2018 (*n* = 12,458).

	CIAD					
	Crude model		Model 1		Model 2	
OBS	95%CI	*P*	95%CI	*P*	95%CI	*P*
Q1	ref		ref		ref	
Q2	0.81(0.70,0.95)	0.01	0.82(0.70,1.46)	0.21	0.90(0.77,1.05)	0.18
Q3	0.71(0.60,0.83)	<0.001	0.72(0.62,0.84)	<0.001	0.83(0.72,0.95)	0.01
Q4	0.68(0.56,0.83)	<0.001	0.71(0.58,0.86)	0.001	0.71(0.64,0.81)	0.01
*p* for trend		<0.001		<0.001		0.01

	COPD					
	Crude model		Model 1		Model 2	
OBS	95%CI	*P*	95%CI	*P*	95%CI	*P*
Q1	ref		ref		ref	
Q2	0.58(0.40,0.84)	0.004	0.60(0.40,1.29)	0.24	0.71(0.46,1.10)	0.12
Q3	0.47(0.33,0.66)	<0.001	0.53(0.37,0.77)	0.001	0.72(0.50,0.93)	0.01
Q4	0.23(0.15,0.36)	<0.001	0.28(0.19,0.43)	<0.001	0.48(0.31,0.77)	0.003
*p* for trend		<0.001		<0.001		0.005

	Asthma					
	Crude model		Model 1		Model 2	
OBS	95%CI	*P*	95%CI	*P*	95%CI	*P*
Q1	ref		ref		ref	
Q2	0.88(0.75,1.05)	0.05	0.90(0.75,0.92)	0.04	0.96(0.76,1.21)	0.55
Q3	0.78(0.66,0.92)	0.01	0.77(0.65,0.90)	0.01	0.84(0.71,0.98)	0.03
Q4	0.69(0.63,0.88)	0.04	0.58(0.49,0.83)	0.01	0.62(0.52,0.73)	0.01
*p* for trend		<0.001		<0.001		<0.001

	Chronic bronchitis					
	Crude model		Model 1		Model 2	
OBS	95%CI	*P*	95%CI	*P*	95%CI	*P*
Q1	ref		ref		ref	
Q2	0.72(0.55,0.96)	0.03	0.73(0.54,1.18)	0.06	0.84(0.62,1.13)	0.23
Q3	0.63(0.51,0.79)	<0.001	0.66(0.53,0.82)	<0.001	0.82(0.67,1.01)	0.06
Q4	0.40(0.28,0.58)	<0.001	0.43(0.29,0.63)	<0.001	0.64(0.44,0.92)	0.02
*p* for trend		<0.001		<0.001		0.01

OBS, Oxidative Balance Score; COPD, chronic obstructive pulmonary disease. Crude Model was unadjusted; Model 1 was adjusted for gender (female or male), age (<40, 40–60 or >60) and race (Mexican American, Non-Hispanic Black, Non-Hispanic White or other races); Model 2 adjusted for Model 1 plus education level (completed high school, less than high school or more than high school), family income-to-poverty ratio (<1.3, 1.3–3.5 or >3.5), smoking status (never smoker, former smoker, or current smoker), drinking status (current heavier drinker, current light/moderate drinker, former drinker or never drinker), marital status (married/living with partner, never married or widowed/divorced), BMI (<25.0, 25.0–29.9 or >30.0 kg/m^2^), energy intake levels, histories of hypertension (yes or no), hyperlipidemia (yes or no), cardiovascular disease (yes or no) and diabetes (yes or no).

The data in Table 4 reveals a notable inverse correlation between higher OBS and the incidence of total CIAD across multiple demographic groups. Notably, this relationship was evident among individuals aged 40 and above, females or males, those with a BMI >30.0 kg/m^2^, Mexican American or Non-Hispanic White, those with married/living with partner, individuals with more than high school, those with current or former smoking and those with a history of diabetes or cardiovascular disease or hypertension, with a statistically significant trend (*p* < 0.05). Nevertheless, stratified analysis did not uncover any significant interactions between OBS and various strata variables (age, sex, ethnicity, education level, family income, marital status, smoking and drinking status, BMI, diabetes, cardiovascular disease, hypertension, and hyperlipidemia) in relation to CIAD prevalence (all p for interaction >0.05) (Table 4). Moreover, our findings indicate a negative association between OBS levels and inflammatory indicators, including C-reactive protein, white blood cells, neutrophil cells and lymphocyte cells (Table 5).

**Table 4:** Stratified analyses of the associations between quartiles of OBS and the prevalence of chronic inflammatory airway diseases (CIAD) in NHANES 2013–2018.

Subgroups	*N*	OBS				*P* for trend	*P* for interaction
		Q1	Q2	Q3	Q4		
Age							0.62
<40	3,870	ref	0.78(0.61,1.01)	0.71(0.54,0.92)	0.82(0.65,1.04)	0.06	
40–60	4,507	ref	0.87(0.67,1.14)	0.73(0.52,1.03)	0.68(0.46,1.02)	0.05	
>60	4,081	ref	0.80(0.62,1.02)	0.70(0.52,0.93)	0.57(0.42,0.77)	<0.001	
Sex							0.80
Male	6,501	ref	0.83(0.64,1.06)	0.76(0.60,0.96)	0.70(0.56,0.87)	0.002	
Female	5,957	ref	0.79(0.62,1.01)	0.65(0.53,0.80)	0.66(0.50,0.85)	<0.001	
Family PIR							0.16
<1.3	2,620	ref	0.72(0.57,0.91)	0.73(0.54,0.98)	0.77(0.60,1.19)	0.14	
1.3–3.5	5,396	ref	0.78(0.59,1.03)	0.75(0.54,1.02)	0.85(0.64,1.13)	0.09	
>3.5	4,442	ref	0.74(0.63,0.85)	0.61(0.76,0.81)	0.69(0.48,1.14)	0.07	
Body mass index							0.26
<25.0 kg/m^2^	3,338	ref	0.85(0.56,1.30)	0.71(0.50,1.01)	0.74(0.52,1.06)	0.07	
25.0–29.9 kg/m^2^	3,979	ref	0.95(0.70,1.29)	0.62(0.47,0.83)	0.83(0.59,1.16)	0.06	
>30.0 kg/m^2^	5,141	ref	0.75(0.60,0.93)	0.80(0.62,1.04)	0.63(0.45,0.86)	0.01	
Race/ethnicity							0.57
Mexican American	1743	ref	0.62(0.35,1.09)	0.47(0.30,0.75)	0.56(0.35,0.89)	0.003	
Non-Hispanic Black	2,734	ref	0.94(0.72,1.23)	0.81(0.62,1.05)	0.79(0.56,1.12)	0.06	
Non-Hispanic White	4,897	ref	0.84(0.68,1.05)	0.72(0.58,0.90)	0.71(0.56,0.90)	0.004	
Other races	3,084	ref	0.64(0.46,0.90)	0.65(0.48,0.87)	0.52(0.36,1.17)	0.09	
Marital status							0.10
Married/living with partner	7,487	ref	0.74(0.57,0.97)	0.73(0.57,0.93)	0.65(0.49,0.85)	0.01	
Never married	2,236	ref	0.88(0.66,1.18)	0.90(0.68,1.18)	0.87(0.64,1.19)	0.37	
Widowed/divorced	2,735	ref	1.00(0.71,1.39)	0.54(0.39,0.75)	0.75(0.53,1.07)	0.06	
Education level							0.11
Completed high school	2,865	ref	1.01(0.73,1.40)	0.93(0.64,1.35)	0.65(0.44,1.28)	0.19	
Less than high school	2,358	ref	0.81(0.56,1.20)	0.43(0.31,0.60)	0.66(0.34,1.23)	0.13	
More than high school	7,235	ref	0.75(0.59,0.96)	0.70(0.57,0.87)	0.75(0.59,1.24)	0.02	
Smoking status							0.21
Former	3,025	ref	0.95(0.68,1.32)	0.75(0.53,1.06)	0.61(0.43,0.86)	0.004	
Now	2,311	ref	0.76(0.57,1.01)	0.77(0.57,1.03)	0.43(0.30,0.63)	<0.001	
Never	7,122	ref	0.89(0.67,1.17)	0.82(0.65,1.02)	1.05(0.83,1.33)	0.78	
Drinking status							0.94
Current heavier drinker	2,660	ref	0.81(0.53,1.24)	0.78(0.63,1.28)	0.79(0.53,1.01)	0.15	
Current light/moderate drinker	7,238	ref	0.87(0.68,0.91)	0.80(0.61,0.85)	0.87(0.61,1.11)	0.20	
Former	1,240	ref	0.94(0.65,0.98)	0.81(0.50,0.92)	1.07(0.71,1.18)	0.10	
Never	1,320	ref	0.89(0.50,0.99)	0.72(0.45,0.84)	0.79(0.61,1.02)	0.17	
DM							0.50
Yes	2,482	ref	0.83(0.60,1.15)	0.82(0.54,1.25)	0.60(0.40,0.89)	0.04	
No	9,976	ref	0.82(0.69,0.97)	0.70(0.58,0.83)	0.72(0.59,1.18)	0.76	
CVD							0.34
Yes	1,396	ref	1.00(0.68,1.47)	0.89(0.56,1.42)	0.46(0.27,0.77)	0.02	
No	11,062	ref	0.83(0.71,0.97)	0.73(0.62,0.86)	0.77(0.64,1.23)	0.22	
Hypertension							0.46
Yes	5,606	ref	0.94(0.74,1.19)	0.80(0.61,1.05)	0.72(0.54,0.97)	0.03	
No	6,852	ref	0.73(0.57,0.93)	0.66(0.52,0.83)	0.69(0.55,1.27)	0.06	
Hyperlipidemia							0.77
Yes	8,323	ref	0.90(0.75,1.07)	0.82(0.66,0.92)	0.90(0.64,1.21)	0.12	
No	4,135	ref	0.89(0.57,1.10)	0.75(0.65,0.82)	0.74(0.55,1.19)	0.16	

OBS, Oxidative Balance Score; PIR, poverty income ratio; CVD, cardiovascular disease; DM, diabetes mellitus. Analyses were adjusted for covariates gender (female or male), age (<40, 40–60 or >60) and race (Mexican American, Non-Hispanic Black, Non-Hispanic White or other races); education level (completed high school, less than high school or more than high school), family income-to-poverty ratio (<1.3, 1.3–3.5 or >3.5), smoking status (never smoker, former smoker, or current smoker), drinking status (current heavier drinker, current light/moderate drinker, former drinker or never drinker), marital status (married/living with partner, never married or widowed/divorced), BMI (<25.0, 25.0–29.9 or >30.0 kg/m^2^), energy intake levels, histories of hypertension (yes or no), hyperlipidemia (yes or no), cardiovascular disease (yes or no) and diabetes (yes or no) when they were not the strata variables.

**Table 5:** Multiple linear regression associations of OBS with inflammatory markers in adults.

Outcomes	*β*(95%CI)	*P*-value
White blood cells	−0.256(−0.352,−0.023)	<0.001
Neutrophil cells	−0.056(−0.117,−0.012)	<0.001
Lymphocyte cells	−0.114(−0.032,−0.326)	0.01
C-reactive protein	−0.074(−0.136,−0.012)	<0.001

OBS, Oxidative Balance Score. Model were adjusted for gender (female or male), age (<40, 40–60 or >60) and race (Mexican American, Non-Hispanic Black, Non-Hispanic White or other races); education level (completed high school, less than high school or more than high school), family income-to-poverty ratio (<1.3, 1.3–3.5 or >3.5), smoking status (never smoker, former smoker, or current smoker), drinking status (current heavier drinker, current light/moderate drinker, former drinker or never drinker), marital status (married/living with partner, never married or widowed/divorced), BMI (<25.0, 25.0–29.9 or >30.0 kg/m^2^), energy intake levels, histories of hypertension (yes or no), hyperlipidemia (yes or no), cardiovascular disease (yes or no) and diabetes (yes or no).

### Mortality analysis

**Figure 3:**
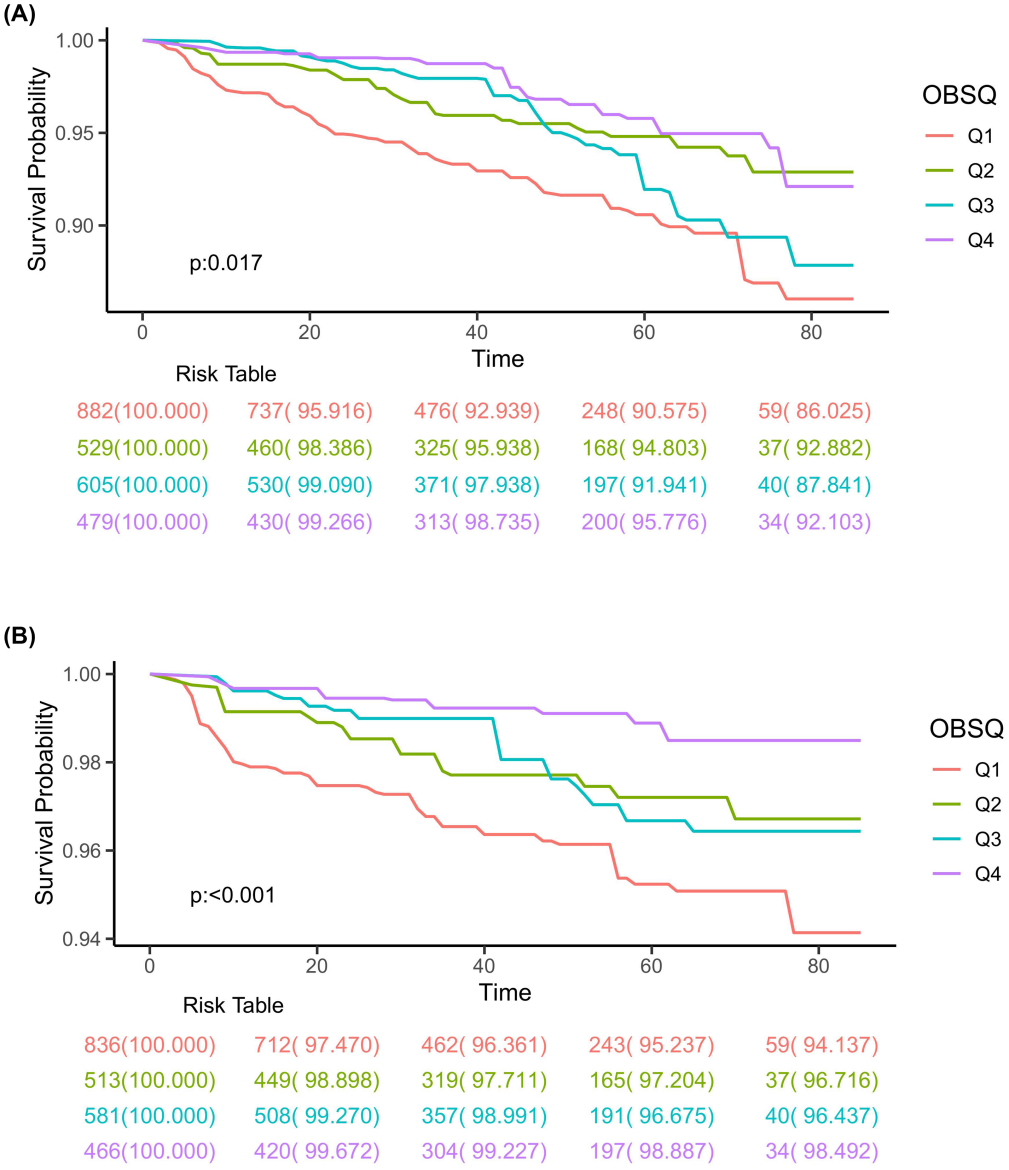
Kaplan–Meier survival curves for all-cause mortality **(A)** and respiratory disease mortality **(B)** in adults with chronic inflammatory airway diseases (CIAD) categorized by quartiles of Oxidative Balance Score (OBS) levels. Quartiles of OBS levels were 3 to 15 (Q1), 16 to 20 (Q2), 21 to 26 (Q3), and 27 to 37 (Q4), respectively.

**Figure 4:**
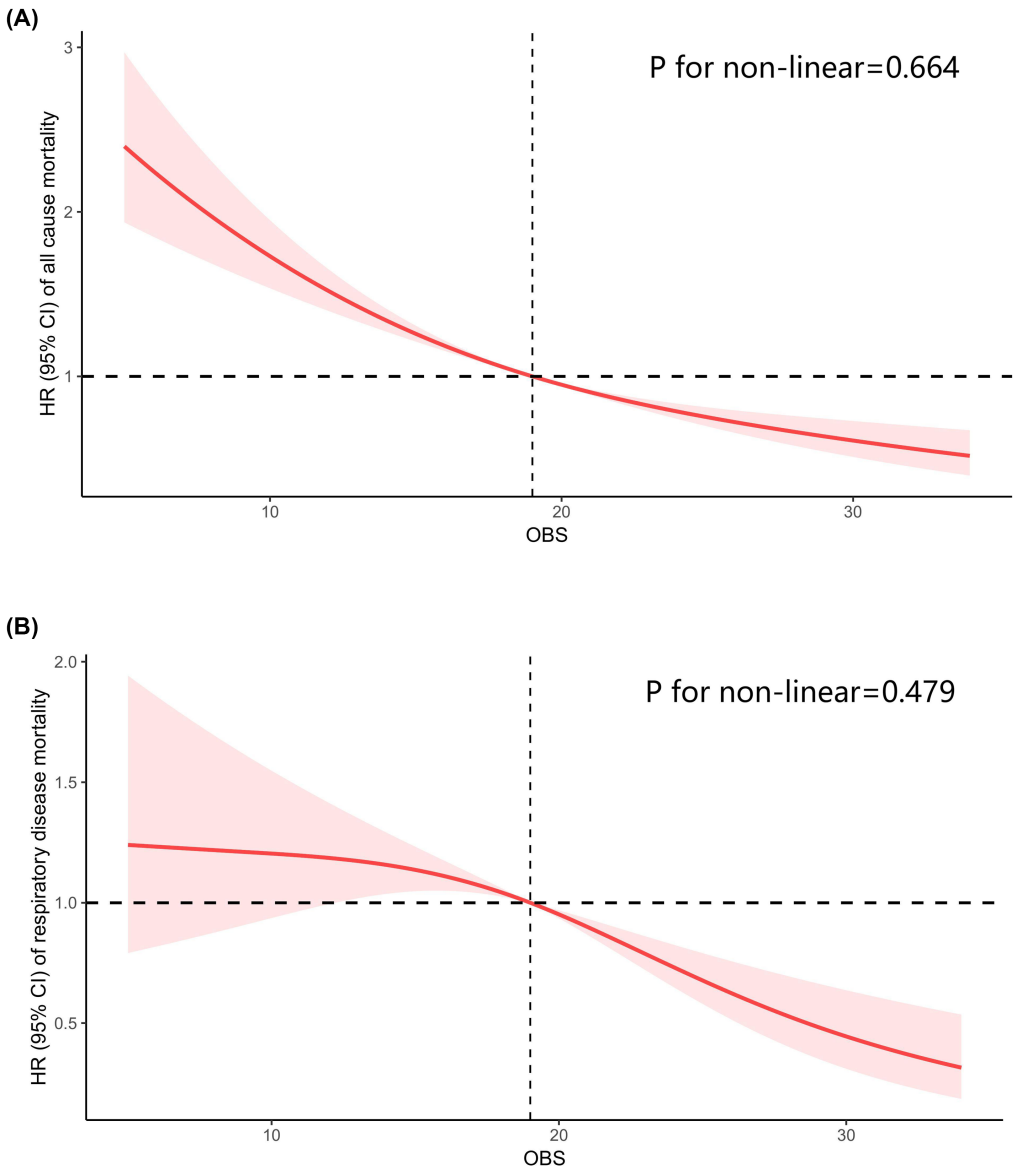
Restricted cubic spline analyses the relationship of Oxidative Balance Score (OBS) levels and the risk of all-cause mortality **(A)** and respiratory diseases mortality **(B)** in adults with chronic inflammatory airway diseases (CIAD). Adjusted for gender (female or male), age (<40, 40–60 or >60) and race (Mexican American, Non-Hispanic Black, Non-Hispanic White or other races); education level (completed high school, less than high school or more than high school), family income-to-poverty ratio (<1.3, 1.3–3.5 or >3.5), smoking status (never smoker, former smoker, or current smoker), drinking status (current heavier drinker, current light/moderate drinker, former drinker or never drinker), marital status (married/living with partner, never married or widowed/divorced), BMI (<25.0, 25.0–29.9 or >30.0 kg/m^2^), energy intake levels, histories of hypertension (yes or no), hyperlipidemia (yes or no), cardiovascular disease (yes or no) and diabetes (yes or no).

Totally, 2,495 individuals with CIAD were included in the research. Over a median follow-up of 3.76 years, there were a total of 169 deaths (6.77%) from any causes and 70 deaths (2.92%) specifically due to respiratory ailments. Kaplan–Meier survival analysis showed that individuals in the highest quartile of OBS had the lowest risk of both all-cause mortality (log-rank test *p* = 0.017; Figure 3A) and respiratory disease mortality (log-rank test *p* < 0.001; Figure 3B). Even after adjusting for multiple factors, individuals in the fourth quartile continued to showed a significantly reduced risk of all-cause mortality (HR = 0.71, 95% CI 0.55–0.93) and respiratory disease mortality (HR = 0.53, 95% CI 0.43–0.74) in comparison to those in the lowest quartile of OBS (Table 6). Furthermore, we observed clear linear relationships between OBS levels and the risks of all-cause mortality (*P* for non-linear = 0.664, Figure 4A) and mortality from respiratory diseases (*P* for non-linear = 0.479, Figure 4B).

**Table 6:** Relationships of OBS with all-cause and respiratory disease mortality in patients with CIAD from the NHANES 2013–2018 (*n* = 2,495).

	All-cause mortality					
	Crude model		Model 1		Model 2	
OBS	95%CI	*P*	95%CI	*P*	95%CI	*P*
Q1	ref		ref		ref	
Q2	0.63(0.50,0.80)	<0.001	0.68(0.53,0.87)	0.002	0.67(0.40,1.20)	0.19
Q3	0.72(0.56,0.92)	0.01	0.84(0.64,1.10)	0.20	0.69(0.50,1.18)	0.23
Q4	0.47(0.33,0.67)	<0.001	0.57(0.39,0.84)	0.004	0.71(0.55,0.93)	0.01
*P* for trend		<0.0001		0.01		0.01

	Respiratory disease mortality					
	Crude model		Model 1		Model 2	
OBS	95%CI	*P*	95%CI	*P*	95%CI	*P*
Q1	ref		ref		ref	
Q2	0.48(0.25,0.84)	0.01	0.52(0.33,0.81)	0.02	0.57(0.34,0.78)	0.01
Q3	0.55(0.27,0.76)	0.01	0.76(0.34,0.90)	0.03	0.60(0.33,1.22)	0.19
Q4	0.47(0.27,0.80)	<0.001	0.50(0.38,0.76)	<0.001	0.53(0.43,0.74)	0.002
*P* for trend		<0.001		0.003		0.01

OBS, Oxidative Balance Score; CIAD, chronic inflammatory airway diseases. Crude Model was unadjusted; Model 1 was adjusted for gender (female or male), age (<40, 40–60 or >60) and race (Mexican American, Non-Hispanic Black, Non-Hispanic White or other races); Model 2 adjusted for Model 1 plus education level (completed high school, less than high school or more than high school), family income-to-poverty ratio (<1.3, 1.3–3.5 or >3.5), smoking status (never smoker, former smoker, or current smoker), drinking status (current heavier drinker, current light/moderate drinker, former drinker or never drinker), marital status (married/living with partner, never married or widowed/divorced), BMI (<25.0, 25.0–29.9 or >30.0 kg/m^2^), energy intake levels, histories of hypertension (yes or no), hyperlipidemia (yes or no), cardiovascular disease (yes or no) and diabetes (yes or no).

## Discussion

Analyzing data from the NHANES 2013–2018, our study explored the connection between OBS and the prevalence of total and specific chronic inflammatory airway diseases (including asthma, chronic bronchitis, and COPD), as well as mortality from all causes and respiratory diseases in adults suffering from CIAD. The findings revealed that individuals in the highest quartile of OBS were significantly linked to a decreased prevalence of total and specific CIAD when adjusting for all potential confounding factors. Additionally, higher OBS levels were associated with reduced mortality from both all causes and respiratory diseases among CIAD patients. The results indicate that adhering to a diet rich in antioxidants, characterized by high consumption of fiber, vitamins, and minerals, and adopting a healthy lifestyle, marked by limiting alcohol and nicotine consumption, can play a significant role in preventing CIAD in adults.

Oxidative stress is the key driving mechanism of CIAD, which promotes the occurrence and development of diseases by damaging cellular DNA, reducing antiprotease activity, and accelerating small airway fibrosis (9). Therefore, maintaining a state of oxidative and antioxidant equilibrium is extremely important to prevent the occurrence of CIAD and delay its progression. However, the respiratory system is at higher risk for oxidative stress due to its direct exposure to the external environment, leading to increased susceptibility to chronic inflammatory airway diseases like asthma, COPD, and chronic bronchitis (9). Although recent studies acknowledge the significance of oxidative stress in these conditions, they have not yet established a clear quantified relationship between oxidative stress and CIAD (25, 26). In this context, OBS emerges as a crucial metric, providing a measurable indicator to assess oxidative imbalance in individuals.

Our research findings regarding OBS and CIAD are dependable. In our investigation, we included 20 factors related to oxidative stress, the majority of which are supported by existing literature indicating their involvement in oxidative stress. In terms of dietary components, intake of dietary fiber may lower the occurrence of CAID (including asthma, chronic bronchitis, and COPD) (27). A diet rich in fiber has been proposed to lessen innate immune-mediated systemic and pulmonary inflammation by the way of gut-lung axis (28). Vitamins including beta-carotene, vitamin C, and vitamin E demonstrate anti-inflammatory and antioxidant properties and are advantageous for lung health, offering protection against chronic bronchitis and COPD (29, 30). Intake of iron, calcium, and selenium is positively associated to indicators of lung function (31). Insufficient magnesium consumption could be linked to the development and progression of COPD (32). An increase in n-6 polyunsaturated fatty acids consumption and a decrease in n-3 polyunsaturated fatty acids consumption can trigger a pulmonary inflammatory response (33). Consistent with our research, adhering to a diet that is high in quality and rich in antioxidants, such as the Mediterranean diet or DASH diet, has been proven to provide a defense against CIAD (34–36).

Regarding lifestyle, it is thought to have a strong connection to the onset and progression of numerous health conditions. Unhealthy lifestyle habits like lack of physical activity, smoking, and excessive alcohol consumption can disrupt body’s oxidative stress process (37, 38), leading to the production of harmful reactive oxygen species which in turn damage airway structural cells (39, 40), cause airway restructuring and reduce lung function (41). A multi-cohort study has shown that maintaining a normal BMI, never smoking, engaging in physical activity, and consuming alcohol in moderation can lower the risk of COPD (42). Moreover, studies have demonstrated that engaging in physical activity can boost the level of antioxidants in muscles, which may potentially enhance muscle performance and shield against inflammation and oxidative stress (43), thereby lowering the likelihood of developing asthma and COPD (44, 45). Conversely, adopting a sedentary lifestyle was associated to a higher prevalence of CIAD (46). Furthermore, smoking is widely recognized as a major contributor to the development of chronic respiratory illnesses (40). Research has consistently shown that cigarette smoke plays a key role in the progression of chronic bronchitis and COPD. Exposure to cigarette smoke triggers the production of free radicals, which can disrupt the normal functioning of mitochondria, ultimately leading to accelerated cell death in lung tissue (47). However, ceasing to smoke can facilitate the restoration of lung function in individuals suffering from CIAD, such as COPD and asthma. In addition, excessive alcohol consumption has also been associated with an increased risk of COPD (39), as well as asthma (48). Alcohol-induced lung injury may arise from various ways such as heightened oxidative stress, changes in tissue structure, and disrupted lung inflammation regulation (41). Recent research has been increasingly endorsing the idea that the best recommendation for alcohol intake is abstaining from drinking (49). Additionally, persistent oxidative stress can activate pathways related to inflammation, leading to the release of inflammatory mediators. The higher levels of these pro-inflammatory mediators can draw more neutrophils and other inflammatory cells, sustaining lung inflammation in individuals with CIAD (47, 50). Studies have shown that levels of inflammation are significantly higher in obese individuals compared to healthy individuals, and obese individuals with asthma may have impaired macrophage/monocytic function, suggesting that obesity could also pose a risk for asthma and COPD (44, 51). A recent study utilizing the NHANES database revealed a connection between obesity and an elevated risk of CIAD, aligning with our own findings (52).

In this study, we further investigated the correlation between OBS and mortality rates from all causes and respiratory diseases in patients with CIAD. To date, research on the relationship between OBS and mortality rates from all cause and specific disease remains limited. Previous research has indicated a connection between OBS and the risk of death from all cause, but these studies were constrained to particular groups and did not cover all pertinent factors comprehensively. For instance, the study by Van Hoydonck et al. (53) focused on male smokers, Kong et al. (54) examined individuals with a high cardiovascular disease risk, and Mao et al. (55) conducted a comparable study involving older women in Iowa. Additionally, some researches (56, 57) have found a negative correlation between OBS and overall mortality in populations with conditions like diabetes or metabolic syndrome, yet all these studies failed to examine the impact of OBS on patients with CIAD specifically. Given the susceptibility of CIAD patients to oxidative stress-related damage, examining the association between OBS and mortality risk in this group holds significant importance in both clinical and public health realms. Utilizing the NHANES database, our study benefits from a large number of observational data and long-term follow-up, taking full advantage of its inclusion of diverse ethnic backgrounds, different educational levels, etc., thereby contributing to the existing evidence base.

The major strength of this research lies in its large, representative sample size, which allowed us to uncover the correlation between OBS and the prevalence of total and specific CIAD, as well as mortality rates from all causes and respiratory diseases in adults. Secondly, the research was adjusted for the primary factors that influence lung function during the analysis, encompassing age, gender, smoking status, BMI, ethnicity, and other potential variables. Our results remained consistent even after accounting for all these variables. Thirdly, the research employed the RCS model to evaluate the dose–response relationship between OBS levels and chronic inflammatory airway diseases, revealing a linear correlation between the two. Finally, our research also explored the link between OBS and the risk of mortality from all causes and respiratory diseases in individuals with CIAD, providing a new perspective that diverges from previous investigations.

Our study also has several limitations that should be noted. Initially, using self-reported data for CIAD diagnosis and collecting dietary data through the 24-h dietary recall method can introduce potential for recall bias. Furthermore, OBS was only assessed at the beginning of the study, which means that any changes or fluctuations in OBS over time were not taken into account. Consequently, our analysis cannot address how OBS may vary longitudinally. To address the constraint of only measuring OBS at the start, future research could consider conducting prospective cohort studies that measure OBS at regular intervals to track its changes over time and its long-term effects on health outcomes. Additionally, our findings cannot establish causal relationships due to the observational study design. In analysis, we made efforts to consider various factors that could influence the relationship between OBS and CIAD, encompassing age, sex, race, PIR, marital status, education level, smoking and drinking habits, and medical history. However, despite adjusting for all these variables, there may still be residual confounding factors. Lastly, it is important to recognize that our study was conducted among CIAD patients in the US, including a diverse sample of participants. While the study involved 1,743 Mexican Americans, 2,734 Non-Hispanic Black individuals, 4,897 Non-Hispanic White individuals, and 3,084 individuals of other races, the generalizability of our findings to other populations may be limited. Therefore, further longitudinal studies with comprehensive factors adjusted are necessary to elucidate the causal mechanisms and validate the long-term impact of OBS on CIAD.

## Conclusion

The results of our study revealed that people with higher OBS tended a lower prevalence of CIAD, including asthma, chronic bronchitis, and COPD. Furthermore, in those with CIAD, higher OBS were linked to a decreased risk of death from all causes and respiratory diseases. These discoveries offer valuable information on the role of diet and lifestyle in preventing CIAD. To validate these results, future investigation should focus on establishing cause-and-effect connections and exploring potential mechanisms, including broader populations and longitudinal data.

## Data Availability

The original contributions presented in the study are included in the article/supplementary material, further inquiries can be directed to the corresponding author.

## Footnotes

^1^ https://www.cdc.gov/nchs/nhanes/index.htm
